# Time-varying risks of infection in patients as they proceed through the phases of ‘pre-RA’: results from the Scottish Early RA inception cohort

**DOI:** 10.1136/rmdopen-2023-003224

**Published:** 2023-11-23

**Authors:** Duncan Porter, Sahil Jain, Evelyn Qian, Fraser R Morton, Iain B McInnes

**Affiliations:** 1 School of Infection and Immunity, University of Glasgow, Glasgow, UK; 2 Gartnavel General Hospital, Glasgow, UK; 3 MVLS College Office, University of Glasgow, Glasgow, UK

**Keywords:** Rheumatoid Arthritis, Infections, Arthritis, Rheumatoid

## Abstract

Rheumatoid arthritis (RA) develops after progressing through sequential ‘pre-RA’ phases. The mechanisms driving progression from one phase to the next remain poorly understood. This study examined the longitudinal rates of community and hospital infections in patients during sequential stages of pre-RA and early arthritis.

**Methods:**

The Scottish Early RA inception cohort recruited patients with newly diagnosed RA. Incidences of infection were determined from community antibiotic prescriptions and serious infections were determined by hospital discharge coding. Dates of diagnosis and symptom onset allowed identification of asymptomatic/symptomatic pre-RA and early arthritis eras to analyse infection rates over time compared with age- and sex-matched controls.

**Results:**

The incidence rate ratio (IRR) seen in the period 0–6 months prior to symptom onset was 1.28 (95% CI 1.15 to 1.42). In ‘symptomatic pre-RA’, the IRR was 1.33 (95% CI 1.18 to 1.49) which persisted into ‘early arthritis’. The rate of hospital admissions was numerically greater in ‘pre-RA’ and significantly greater in ‘early arthritis’ (IRR 1.82, 95% CI 1.32 to 2.46).

**Conclusion:**

Antibiotic risk is increased in patients with ‘pre-RA’ at least 6 months before symptoms develop, and this persists throughout the symptomatic pre-RA phase. Infections may be important in the mechanisms that drive progression to RA or be a manifestation of immune dysfunction (or both). These observations could inform safety and efficacy considerations for interventions in pre-RA to prevent progression. Patients with ‘pre-RA’ with recurrent antibiotic use may also be an identifiable ‘high risk’ group that could enrich the study population for intervention studies in pre-RA.

**WHAT IS ALREADY KNOWN ON THIS TOPIC**

- In the development of rheumatoid arthritis (RA), the pre-RA phases can be broadly divided into ‘asymptomatic pre-RA’, ‘symptomatic pre-RA’ and ‘early arthritis’ phases.
- In these ‘pre-RA’ phases, immunological dysregulation is detectable, and increasingly, psychological and physical morbidity.

**WHAT THIS STUDY ADDS**

- In this study we find a rise in antibiotic use, probably reflecting an increased infection rate starting at least 6 months prior to the development of symptoms compared with controls.
- This suggests that infections (and/or antibiotic use) may be important in the mechanisms that drive progression from pre-RA phases to clinical RA.

**HOW THIS STUDY MIGHT AFFECT RESEARCH, PRACTICE OR POLICY**

- There may be implications for the safety and efficacy of interventions in pre-RA to reduce the likelihood of progression to RA.
- Individuals who require recurrent use of antibiotics could be a useful ‘higher risk’ group of patients that could be used to enrich the study population in intervention studies in pre-RA.

## Introduction

Rheumatoid arthritis (RA) develops after a prodromal phase, characterised by immunological dysregulation, followed by progressive emergence of clinically detectable disease. EULAR describes people ‘at-risk’ of developing RA in six stages:

- Genetic risk factors for RA (eg, shared epitope)
- Environmental risk factors for RA (eg, cigarette smoking)
- Systemic autoimmunity (eg, presence of autoantibodies)
- Symptoms without classical arthritis
- Unclassified arthritis and
- RA.^1^

In patients who develop RA, stages (a)–(e) can be referred to as a ‘pre-RA’ state: stages (a)–(c) are asymptomatic, and stages (d)–(e) make up the symptomatic pre-RA phase. Not all patients exhibit all pre-RA stages, and the development of RA is a dynamic process that may not be homogeneous across the RA population.^2^ Serological studies have demonstrated that autoantibodies (such as rheumatoid factor and anti-citrullinated protein antibodies (ACPAs)) may be present for several years before symptoms develop. The concentration of ACPA rises, and there is evidence of epitope spreading in the period immediately preceding the development of overt synovitis.^3^ However, the mechanisms that drive the transition from one pre-RA state to another are poorly understood. A better understanding of those mechanisms has the potential to inform future therapeutic approaches to slow or stop the transition from pre-RA to RA.

RA is associated with an increase in all-cause and infection-related mortality. In population-based inception cohorts, it is estimated that there is an increased risk of infections requiring hospitalisation compared with age- and sex-matched controls (adjusted HR 1.83 (95% CI 1.52 to 2.21))^4^ with an approximately fourfold to sixfold increase in infection-related mortality.^5^ The explanation for these observations is complex and is likely to be multifactorial. Risk factors such as disease-related immune dysfunction, age, disease activity, comorbidity and medication may be relevant, but it is challenging to assess the relative contribution of each to the overall infection risk.^6^ Assessing the risk of infection in the different phases of pre-RA may be instructive in elucidating the relative contribution of treatment (not yet started) and immune dysfunction.

Previous studies of the overall incidence of infection in pre-RA have shown conflicting results. Some studies have found an increased infection risk: a small cohort of first-degree relatives of Tatarstan patients who subsequently developed RA had an *increased* rate of infection up to 2 years before their diagnosis.^7^ A large case-controlled cohort in the UK of 8000 patients with RA identified from primary care records showed an *increased* use of antibiotic prior to the diagnosis of RA. There was a ‘dose-dependent’ risk of developing RA that increased with the number of antibiotic courses—the relative risk of developing RA was 2.65 (95% CI 2.40 to 2.93) for those who received more than 10 courses of antibiotics over a 5-year period. Diagnosis was defined as the first date that the diagnostic code was entered into a patient’s record, and there were no data on whether/when the diagnosis had been made by a rheumatologist.^8^ On the other hand, a Swedish inception cohort of 3000 patients with RA found a *reduction* in the incidence of infection in the 2 years prior to the diagnosis compared with controls, using a questionnaire to collect data retrospectively at the time of diagnosis of RA.^9^ A similar study of 400 Canadian patients found a *reduced* likelihood of being prescribed antibiotics in the year preceding diagnosis, compared with rheumatology patients with non-inflammatory conditions such as osteoarthritis, fibromyalgia and tendinitis.^10^

## Methods

The Scottish Early RA (SERA) inception cohort and biobank recruited 1073 patients from across Scotland, UK, with newly diagnosed RA or undifferentiated arthritis between March 2011 and April 2015.^11^ Each patient in Scotland has a unique identifier which allows data linkage with national data on community prescribing, microbiology cultures and hospital admissions. The Prescribing Information System (PIS) provides data on all scripts that are cashed at community pharmacies.^12^ Positive microbiological culture results are available from the Electronic Communication of Surveillance in Scotland database. Hospital admission records for the Scottish population are also available with reasons for admission using ICD-10 codes.^13^

Serious infections were identified from admissions coded with relevant ICD-10 diagnoses as previously described.^14^ The PIS stores data about all drugs and doses prescribed in general practice and other community settings, and the prescription of an antibiotic and antiviral drug was used as a surrogate for community (non-serious) infections. The total number of infections was estimated as the sum of the number of antibiotic scripts plus the number of admissions for serious infection during pre-RA and postdiagnosis stages. Severity was measured using the ratio of serious to total infections.

For each patient, data were collected on the date of symptom onset as recalled by the patient at the time of their inclusion in the study at and date of diagnosis. To study time-varying risks of infection in different phases of pre-RA and early arthritis, data were split into three stages:

1. **Asymptomatic pre-RA phases (a)–(c**)
  - Infection rates were calculated for eras of 6 months duration over 18 months leading up to the date of symptom onset.
2. **Symptomatic pre-RA phase (d**)
  - Infection rates were calculated in the period between the date of symptom onset and the date of diagnosis. Because some patients reported a very long period of symptom duration, the analysis was restricted to those patients with a symptomatic pre-RA phase of <12 months.
3. **Early arthritis phase (e)–(f**)
  - Infection rates were calculated for eras of 6 months duration over 18 months following the diagnosis of undifferentiated arthritis (UA) or RA. The infection rates of patients who fulfilled the ACR/EULAR 2010 Classification Criteria at recruitment were compared with those who had UA and were found to be very similar (data not shown), so the groups were analysed together

Five control individuals, who were alive on the date of the patient’s diagnosis, were identified for each SERA participant and were matched for age, sex and geography (based on partial postcode, thereby partially adjusting for social deprivation) by the electronic Data Research and Innovation Service (eDRIS) team at Public Health Scotland. The rates of infection in SERA participants were compared with those in the matched controls.

### Statistical analysis

The baseline data for patients who experienced no infections were compared with those who had one or more community infections (but no admission) and those who had at least one admission during the study period. The rates of community, inpatient and total infections were calculated for each era and were compared with the rates observed in the control population. R, V.4.0.3, was used for all statistical analyses in this study.^15^ Infection rates and CIs for each outcome variable were calculated for the RA and control participants for all eras using the epi.conf function in the epiR package.^16 17^ The incidence rate ratios (IRRs) between the RA and control participants were calculated using the rate ratio function in the epitools R package.^18^ Comparisons between the proportions of severe infections were performed using the two-proportions z-test.

## Results

The characteristics of the patients at diagnosis are shown in table 1. Patients who had at least one hospital admission with an infection were older (62 years vs 58 years, p<0.02) and more disabled (Health Assessment Questionnaire 1.4 vs 1.1, p<0.002) than patients who had no episodes of infection.

**Table 1:** Baseline data

	All patients n=824	Patients with no courses of antibiotics* n=219	Patients with >1 courses of antibiotics but no infection-related admissions* n=530	Patients with ≥1 infection-related admissions* n=75
Age (years)†	59.1 (13.7)	58.1 (12.9)	59.1 (13.9)	62.0 (14.7)
Sex				
Male	299 (36.3)	86 (39.3)	177 (33.4)	36 (48.0)
Female	525 (63.7)	133 (60.7)	353 (66.6)	39 (52.0)
Smoking status				
Current or previous	505 (61.4)	123 (56.4)	334 (63.1)	48 (64.0)
Never smoked	317 (38.6)	95 (43.6)	195 (36.9)	27 (36.0)
(Missing)	2	1	1	0
Symptom duration (days)‡	131 (82–197)	132 (92.5–198)	131 (80–197.8)	126 (73–185)
DAS28-CRP†	4.8 (1.3)	4.7 (1.4)	4.8 (1.3)	5.1 (1.3)
(Missing)	55	15	33	7
HAQ score† (0–3)	1.2 (0.8)	1.1 (0.8)	1.3 (0.8)	1.4 (0.8)
(Missing)	4	2	2	0
CCP (% positive)	412 (61.3)	108 (60.0)	270 (61.4)	34 (65.4)
(Missing)	152	39	90	23
RF (% positive)	289 (58.3)	74 (56.5)	193 (59.8)	22 (52.4)
(Missing)	328	88	207	33

Data are n (%) unless otherwise stated.

*At any stage during the pre-RA or early arthritis stage.

†Mean (SD).

‡Median (IQR).

CCP, anti-cyclic citrullinated peptide antibodies; DAS28-CRP, 28 Joint Count Disease Activity Score; HAQ, Health Assessment Questionnaire; RA, rheumatoid arthritis ; RF, rheumatoid factor.

**Figure 1:**
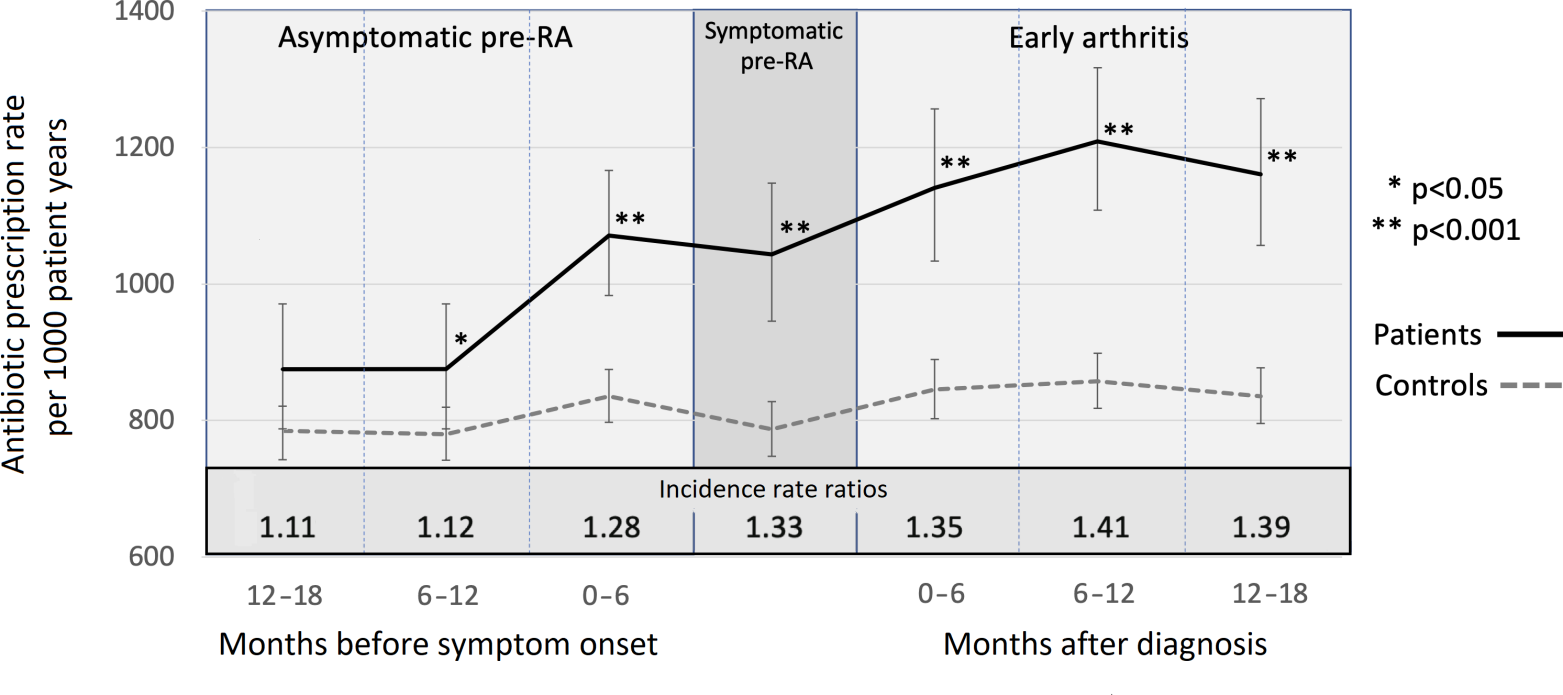
Rate of community antibiotic prescriptions (per 1000 patient-years) and incidence rate ratios in eras during asymptomatic pre-RA, symptomatic pre-RA and early arthritis phases. RA, rheumatoid arthritis.

The rate of community infections was stable throughout the study in the controls at ~800 per 1000 patient-years. The IRR of infection was 1.35–1.41 in the first 18 months of disease when compared with controls (p<0.001). The IRR of infection was also raised (1.33, 95% CI 1.18 to 1.49) in the symptomatic phase of pre-RA. In the asymptomatic phase of pre-RA, there was an increased IRR seen in the period 0–6 months (1.28, 95% CI 1.15 to 1.42) and 6–12 months (1.12, 95% CI 1.00 to 1.26) prior to the development of symptoms (figure 1). There were no differences in the IRR between seropositive (rheumatoid factor (RF) and/or CCP) and seronegative participants (data not shown).

**Figure 2:**
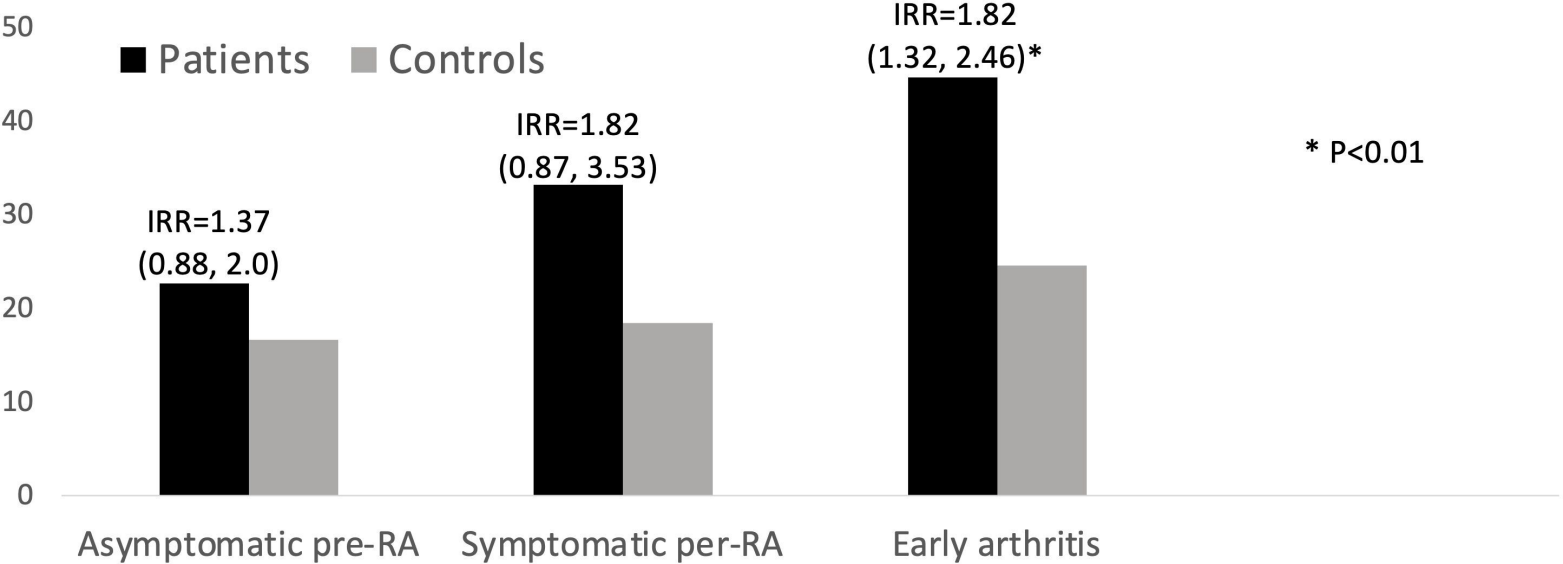
Rate of hospital admissions per 1000 patient-years. IRR, incidence rate ratio; RA, rheumatoid arthritis.

**Figure 3:**
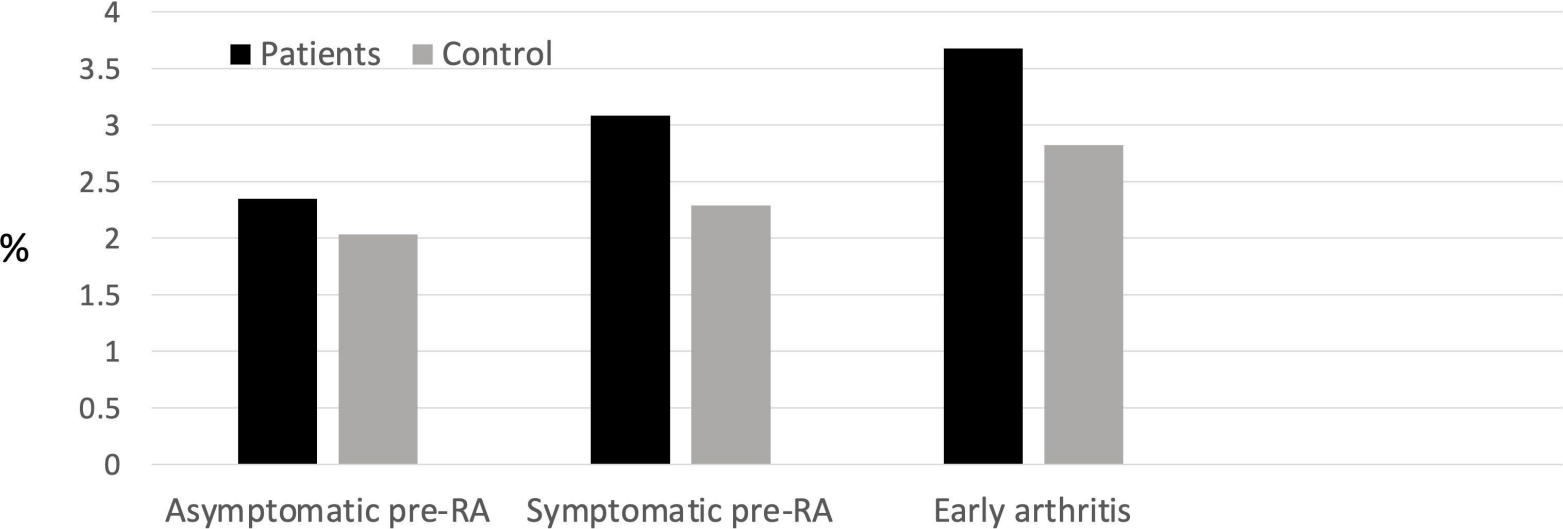
Percentage of infections resulting in hospital admission. RA, rheumatoid arthritis.

The rate of hospital admissions was numerically greater in asymptomatic and symptomatic pre-RA, and significantly greater in early arthritis (IRR 1.82, 95% CI 1.32 to 2.46, figure 2). The percentage of total infections that resulted in hospital admission was not significantly different in patients and controls, or in the different phases of pre-RA/RA ranging from 2% to 3.6% (figure 3). Analysis of the causative organisms grown in bacteriological culture showed that *Escherichia coli* infections were more common in patients when compared with controls (19.6 infections per 1000 patient-years versus 12.5, IRR 1.6, 95% CI 1.15 to 2.1, p=0.003). There were no other significant differences in the rates of positive cultures with other organisms, including *Staphylococcus, Haemophilus, Streptococcus, Proteus, Campylobacter* and *Pseudomonas*.

## Discussion

The model of sequential pre-RA stages provides a very helpful framework for our understanding of the pathogenesis of RA but raises many important questions—for instance, what is the risk of an individual progressing from one stage to another (eg, asymptomatic presence of ACPA to non-specific arthralgia)? Is the process one directional, or can tolerance be re-established leading to the reversal of the process with resolution of synovitis? What, if any, are the consequences of a state of systemic autoimmunity for general health? And what are the factors that promote progression through the different phases?

Immune dysfunction in pre-RA has implications for general health, which may be relevant to the mechanisms driving the transition from pre-RA to RA, either directly or indirectly. Prior studies have explored fatigue, work disability, cardiovascular (CV) and psychiatric disease in pre-RA. For example, in the symptomatic phase of pre-RA, work-related disability and fatigue are present for more than a year before synovitis develops.^19^ RA is associated with an increased risk of CV disease compared with the general population^20^ and one study found that the incidence of acute myocardial infarction was significantly higher in the 2-year period *prior* to the diagnosis of RA when compared with age−sex matched controls (OR 3.17, 95% CI 1.16 to 8.68).^21^ In contrast, a larger, more recent Swedish study of early arthritis patients with a short symptom duration found no increase in CV disease prior to diagnosis^22^ but that this becomes evident within a year of the diagnosis being made.^23^ A higher incidence of psychiatric disease has also been noted in the 3- to 5-year period leading up to the diagnosis of different immune-mediated inflammatory diseases, including RA.^24^ The observation that an increased risk of psychiatric disease occurs so long before the emergence of RA suggests that the risk may exist even in the asymptomatic phases of pre-RA. There are various possible explanations: psychiatric disease and RA may share common aetiological factors; psychiatric illness may predispose to or accelerate the development of overt RA; or there may be sufficient inflammation or disordered immunity in pre-RA to predispose to psychiatric illness—that is, the association between psychiatric disease and the development of RA may be one of confounding, causality or reverse causality.

In this study, there was a rise in the use of antibiotics, which is likely to reflect an increased infection rate prior to the development of RA that extended back at least 6 months prior to the development of symptoms, through the symptomatic pre-RA phases and into the early years after diagnosis. These findings can be interpreted in several ways. The case-controlled study design does not allow conclusions about causality, but several hypotheses are plausible. Direct causality whereby infection results in the development of RA is possible: several putative infectious triggers have been suggested including *Porphyromonas gingivalis*, *Prevotella copri*, *E. coli* and Epstein Barr Virus.^2 25–27^ *Gingivitis*, in particular, is of interest through its link with periodontal disease and has been linked directly to an increase in Peptidylarginine deiminase enzyme activity in producing citrullinated peptides, the target of RA-specific anti-citrullinated peptide antibodies.^2 25 26 28^ The finding by Armstrong *et al*^8^ that the number of antibiotic courses in pre-RA has a ‘dose-dependent’ relationship with the risk of subsequent RA might suggest that it might be the overall burden of infective illness rather than one specific causative organism. The authors of that study postulate that it could be the effects of antibiotic therapy on the gut (or oral) microbiome (rather than the infection itself) that leads to the development of RA.^8 29^ In support of this hypothesis, an altered gut microbiome has been identified in both mouse models and human pre-RA patients.^30–32^ *E. coli* was the most commonly identified pathogen in this study, and it is feasible that this could be the result of emerging gut dysbiosis over time. There is some evidence in mouse models that correction of gut dysbiosis using probiotics may be effective in reducing symptoms^33^ though human studies in patients with RA have been negative.^34^

Reverse causality is also a plausible explanation: dysfunctional immune responses in pre-RA might predispose to an increased use of antibiotics, either because of an increased susceptibility to infection or, potentially, an increase in symptoms that are mistaken for infection. The finding that the increased risk of infection persists into early disease would support the concept of an ongoing infection susceptibility associated with the disease itself (rather than infection being a ‘trigger’ for disease development). Patients with RA have reduced response to vaccinations, but there is also evidence of reduced antibody response to some vaccines when given in the pre-RA phase.^35^ A range of pro-inflammatory cytokines, chemokines and acute phase reactants are elevated in the pre-RA period, with the number of different cytokines that are elevated being a predictor of the interval before RA emerges.^36 37^ The observations that other types of ill health, such as fatigue, cardiovascular and psychiatric disease may also be more prevalent in pre-RA lends credence to the hypothesis that pre-RA is a pathological state that has systemic consequences.

It is possible that both processes are important, and that infections are *both* the catalyst for the development *and* the result of autoimmunity. This could lead to a positive feedback loop whereby infections promote a milieu that promotes the development of autoimmunity, which is associated with an increased susceptibility to infections, generating a self-perpetuating spiral. This might have significant implications for the best strategy to pursue in studies that attempt to slow or prevent the transition from pre-RA to RA; for instance, the use of immune modulators that increase the risk of infection might result in an increased rate of progression. On the other hand, if gut dysbiosis is an important factor in progression, then attempts to normalise the gut microbiome, for instance through diet, might be beneficial if undertaken early enough in the process.

The study has some important strengths and weaknesses. Strengths include the use of an inception cohort recruited from across Scotland, with verification of the diagnosis by a rheumatologist in secondary care. The selection of multiple controls for each patient, matched for age, sex and social deprivation, increased the power of the study. Linkage with national databases allowed the capture of data about community antibiotic prescribing, hospital admissions and microbiology cultures over time periods that preceded the diagnosis. However, there are also limitations to the study; the prescription of antibiotics in the community was used as a surrogate for mild infections, but it is likely that some prescriptions were issued for non-infectious illness (such as fatigue and myalgia which could represent prodromal symptoms of RA). Second, each antibiotic prescription has been used as a surrogate for a discrete infection, but it is likely that sometimes two or more courses of antibiotics might have been prescribed for the same infection. Third, the date of symptom onset was identified through patient recall and is of uncertain accuracy. Fourthly, the analysis assumes that various stages of pre-RA/RA are discrete, whereas in practice it is likely the transition between phases is a gradual process that emerges over time. Lastly, the association between antibiotic use/infection and pre-RA may not be causal but the result of residual confounding. Cases and controls in this study were matched for age, sex and social deprivation but patient-level data on relevant factors such as smoking, diet, employment, obesity and multimorbidity were not available for the controls and so the IRRs could not be adjusted for these factors.

In summary, antibiotic use, probably reflecting an increased rate of infection, is more prevalent in people who subsequently develop RA for at least 6 months in the asymptomatic phase of pre-RA, extending through the symptomatic phase and into early arthritis. Infections (and/or antibiotic use) may, therefore, be important in the mechanisms that drive progression to RA. These observations have implications for the safety and efficacy of interventions that could be tried in pre-RA to reduce the likelihood of progression to RA. It is also possible that the identification of individuals who require recurrent use of antibiotics could be a useful strategy in identifying a group of patients at higher risk of developing RA that could be used to enrich the study population in intervention studies in pre-RA.

10.1136/rmdopen-2023-003224.supp1 Supplementary data

## Data Availability

Data may be obtained from a third party and are not publicly available. SERA data are available for use subject to approval by the Scottish Early Rheumatoid Arthritis Access Committee.
