# Supplementary material for: Time-varying risks of infection in patients as they proceed through the phases of ‘pre-RA’: results from the Scottish Early RA inception cohort
Source: RMD Open. 2023 Nov 23;9(4):e003224. doi: 10.1136/rmdopen-2023-003224 (PMC10668306; doi:10.1136/rmdopen-2023-003224)
Supplement: Supplementary data [file rmdopen-2023-003224supp001.pdf]

Appendix 1: SERA investigators

Steering Committee Members: Duncan Porter[1] and Iain McInnes[1] (Chief Investigators); Stuart H. Ralston[3], Cosimo de Bari[2], John Harvie[5], Carl S. Goodyear[1], Janet Liversidge[2]

Study Team: Caron Paterson[1], Jane Hair[4], Sharon Kean[6], Ashley Gilmour[1]

Investigators: Margaret Duncan, Ayr Hospital; Susan Fraser, Southern General Hospital, Glasgow; Lisa Hutton, Inverclyde Royal Hospital; John Harvie, Raigmore Hospital, Inverness; Vinod Kumar, Ninewells Hospital, Dundee; Mike McMahon, Dumfries & Galloway Royal Infirmary; Robin Munro, Wishaw General Hospital; John Larkin, Victoria Infirmary Glasgow; Neil McKay, Western General Hospital, Edinburgh; John McLaren, Whyteman's Brae Hospital, Fife; David M Reid, Aberdeen Royal Infirmary; Ruth Richmond, Borders General Hospital, Melrose; Gillian Roberts, Vale of Leven Hospital; Sarah Saunders, Glasgow Royal Infirmary and Hilary Wilson, Stobhill Hospital, Glasgow.

1. University of Glasgow, Institute of Infection, Immunity and Inflammation, Glasgow
2. School of Medicine and Dentistry, University of Aberdeen, Aberdeen
3. Rheumatology and Bone Diseases Unit, Centre for Genomic and Experimental Medicine, MRC Institute of Genetics and Molecular Medicine, Western General Hospital, University of Edinburgh
4. NHS Greater Glasgow & Clyde Bio-repository, Pathology Department, Southern General Hospital, Glasgow
5. Forth Valley Royal Hospital, Stirling Rd, Larbert
6. Robertson Centre for Biostatistics, Institute of Health and Wellbeing, University of Glasgow
